# Exploring the Phenotypic Heterogeneity and Bioenergetic Profile of the m.13513G>A mtDNA Substitution: A Heteroplasmy Perspective

**DOI:** 10.3390/ijms26104565

**Published:** 2025-05-10

**Authors:** Tatiana Krylova, Yulia Itkis, Polina Tsygankova, Denis Chistol, Konstantin Lyamzaev, Vyacheslav Tabakov, Svetlana Mikhaylova, Natalia Nikitina, Galina Rudenskaya, Aysylu Murtazina, Tatiana Markova, Natalia Semenova, Natalia Buchinskaya, Elena Saifullina, Hasyanya Aksyanova, Peter Sparber, Natalia Andreeva, Natalia Venediktova, Alina Ivanushkina, Daria Eliseeva, Yulia Murakhovskaya, Natalia Sheremet, Ekaterina Zakharova

**Affiliations:** 1 Research Centre for Medical Genetics, 115522 Moscow, Russia; 2 Belozersky Institute of Physico-Chemical Biology, Lomonosov Moscow State University, 119992 Moscow, Russia; lyamzaev@gmail.com; 3 The “Russian Clinical Research Center for Gerontology” of the Ministry of Healthcare of the Russian Federation, Pirogov Russian National Research Medical University, 129226 Moscow, Russia; 4 Russian Children’s Clinical Hospital, 119571 Moscow, Russia; 5 State Autonomous Healthcare Institution of the Sverdlovsk Region «Clinical and Diagnostic Center ‘‘Maternal and Child Health Protection’’», 620041 Ekaterinburg, Russia; 6 Saint-Petersburg State Medical Diagnostic Center (Genetic Medical Center), 194044 Saint-Petersburg, Russia; 7 Department of Medical Genetics and Fundamental Medicine, Bashkir State Medical University, 450008 Ufa, Russia; 8 State Budgetary Healthcare Institution of Nizhny Novgorod Region «Nizhny Novgorod Regional Children’s Clinical Hospital», 603005 Nizhny Novgorod, Russia; 9 Research Institute of Eye Diseases, 119021 Moscow, Russia; 10 Saint-Petersburg State Budgetary Healthcare Institution City Clinic No. 14, 194021 Saint-Petersburg, Russia; 11 Research Center of Neurology, 125367 Moscow, Russia; ddeliseeva@gmail.com; 12 I.M. Sechenov First Moscow State Medical University, the Department of Ophthalmology, 119991 Moscow, Russia

**Keywords:** mtDNA, heteroplasmy, LHON, Leigh, MELAS, respirometry

## Abstract

The m.13513G>A (p.Asp393Asn) substitution in the *MT-ND5* (Mitochondrially Encoded NADH/Ubiquinone Oxidoreductase Core Subunit 5) gene is a common pathogenic variant associated with primary mitochondrial disorders. It frequently causes Leigh syndrome and mitochondrial encephalomyopathy with lactate acidosis and stroke-like episodes (MELAS). In this study, we present clinical data, heteroplasmy levels in various tissues (blood, urine, and skin fibroblasts), and bioenergetic characteristics from a cohort of 20 unrelated patients carrying the m.13513G>A mutation, classified according to the following phenotypes: Leigh syndrome (*n* = 12), MELAS (*n* = 2), and Leber’s hereditary optic neuropathy (LHON, *n* = 6). We observed a significant correlation between high respiratory ratios and heteroplasmy levels in fibroblast cell lines of the patients. Furthermore, fibroblast cell lines with heteroplasmy levels exceeding 55% exhibited markedly reduced mitochondrial membrane potential. These findings contribute to a better understanding of the clinical and bioenergetic profiles of patients with m.13513G>A-variant-related phenotypes across different heteroplasmy levels, based on data from a single genetic center. Our data suggest that even a slight shift in heteroplasmy can improve cellular function and, consequently, the patients’ phenotype, providing a solid foundation for the development of future gene therapies for mtDNA diseases.

## 1. Introduction

Primary mitochondrial diseases (PMDs) may be defined as a heterogeneous group of inborn errors of metabolism. They are caused by structural and/or functional abnormalities of the oxidative phosphorylation (OXPHOS) system, which are due to pathogenic variants in both mitochondrial (mtDNA) and nuclear (nDNA) DNA [1].

Mitochondria are essential for cellular energy production, primarily through OXPHOS, a process that involves a series of protein complexes embedded in the inner mitochondrial membrane [2]. Among these, complex I (NADH: ubiquinone oxidoreductase) is the largest and most intricate, consisting of forty-five subunits, seven of which are encoded by mtDNA and the remaining thirty-eight by nDNA. The initial step in the electron transport chain is catalyzed by complex I, whereby electrons are transferred from NADH to ubiquinone. This is coupled with proton translocation across the inner mitochondrial membrane, generating the proton gradient essential for ATP synthesis [3].

Isolated complex I (CI) deficiency represents the most prevalent disorder within the OXPHOS system, manifesting in a wide range of clinical phenotypes [4,5]. The clinical presentation of CI deficiency is highly variable, indicative of the underlying genetic heterogeneity due to pathogenic variants in either nDNA or mtDNA, and from mtDNA heteroplasmy—a unique phenomenon of mtDNA, characterized by the coexistence of both wild-type and mutant mtDNA within the same cell [6]. To date, approximately 38 confirmed pathogenic variants across seven mtDNA-genes-encoding structural subunits of CI (*MT-ND1*, *MT-ND2*, *MT-ND3*, *MT-ND4*, *MT-ND4L*, *MT-ND5*, and *MT-ND6*) have been determined to be associated with various PMDs [7,8]. One of the most well-known mtDNA variants is m.13094T>C in the *MT-ND5* gene. A wide clinical spectrum, ranging from Leigh syndrome to LHON, has been shown to be clearly associated with different mutation loads observed in patient’s cells [9].

Among different mtDNA point mutations, the m.13513G>A substitution in the *MT-ND5* gene, which results in an amino acid substitution from aspartic acid to asparagine at position 393 (p.Asp393Asn) in the ND5 subunit of complex I, is of particular note. This variant was initially described by Santorelli and was identified in a patient with MELAS syndrome in heteroplasmy [10]. To date, this substitution has been implicated in a range of mitochondrial phenotypes, including LHON, MELAS, and Leigh syndromes, often correlated with varying degrees of heteroplasmy, with over 50 reported cases from different medical centers in the literature [11]. The phenotypic expression of the m.13513G>A mutation is highly variable, even among individuals with similar levels of heteroplasmy, suggesting the potential influence of additional genetic, environmental, or epigenetic factors.

It is noteworthy that all pathogenic variants in the *MT-ND5* gene are likely to exert detrimental effects, even at very low mutation loads [9,12].

The connection between low mutation loads in the *MT-ND5* gene and disease phenotype can be attributed to the critical role of ND5 in mitochondrial complex I: ND5 is situated in close proximity to the main site of reactive oxygen species (ROS) production within the mitochondria and is crucial for the proper assembly and functioning of the complex I [13].

Our study presents clinical and molecular data from 20 patients with m.13513G>A substitution at one center, demonstrating a range of phenotypes: LHON (N = 6), MELAS (N = 2), and Leigh syndrome (N = 12). Additionally, we investigated the association between heteroplasmy levels in different tissues and the clinical and bioenergetic features of these patients.

## 2. Results

### 2.1. Clinical Data

**Figure 1:**
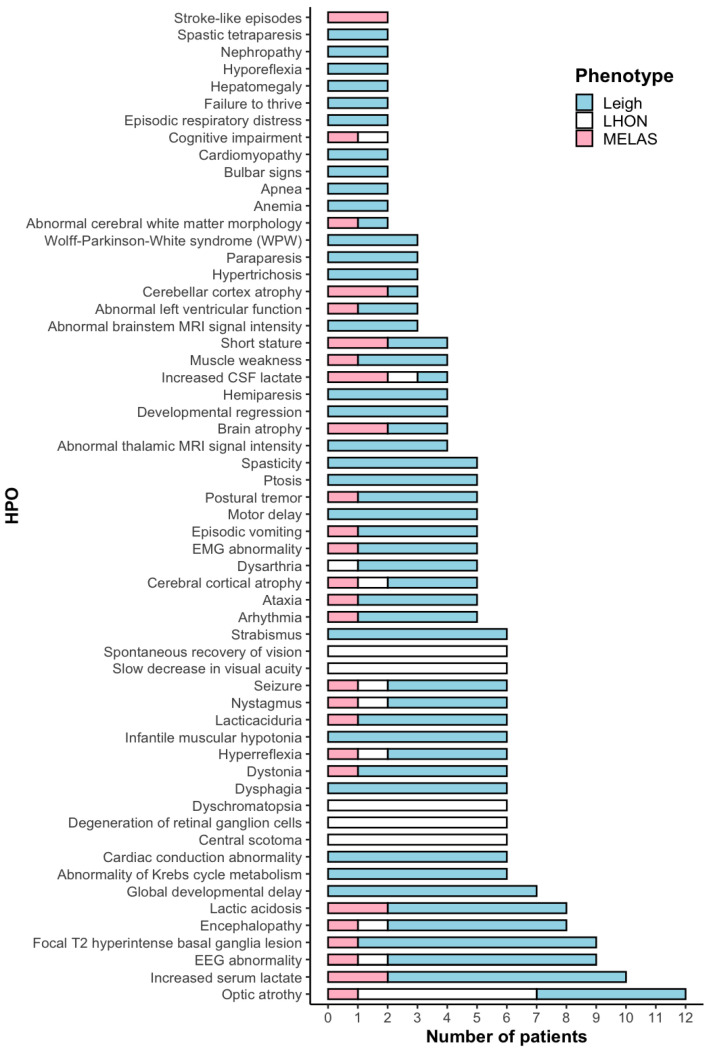
HPO terms in 17 patients with m.13513G>A variant.

Clinical data were available for 17 out of 20 unrelated patients, including those with Leigh syndrome (*n* = 9), MELAS (*n* = 2), and LHON (*n* = 6, Patient 16 was described previously) [14]. The average age of onset for the Leigh group was 1.42 years (range: 0 months to 11 years). Figure 1 illustrates the primary clinical features of these patients based on 59 HPO terms [15,16]. Elevated lactate concentrations, either in serum or cerebrospinal fluid, were observed in 11 out of 20 patients (Table S1). Lesions in the basal ganglia were the most frequent clinical feature, present in 9 out of 11 Leigh and MELAS patients.

Among patients with heteroplasmy levels exceeding 69% in blood, mortality due to multiple organ failure syndrome was observed in three cases (before 1 year of age in two patients and at 8 years of age in one patient). Early disease manifestation (before 1 year of age) was more common in patients with blood heteroplasmy levels above 67%. In our cohort, 3 patients were diagnosed with Wolff–Parkinson–White (WPW) syndrome.

The average age of onset in our LHON cohort was 18.1 years (Table S2). The initial symptoms typically involved a bilateral, painless loss of vision, with the second eye becoming affected within up to 3 weeks. No additional symptoms were observed in the patients, except for Patient 20, who had a demyelinating disorder [17]. Visual acuity at its nadir ranged from 0.005 to 0.08, based on a Sitsev–Golovin table. MRI scans were normal for all patients except Patient 20. All patients experienced a spontaneous recovery of visual acuity, with improvements ranging from 0.1 to 1.0. Patient 19 was unable to undergo a formal examination but reported subjective improvement in vision. Despite the improvement in visual acuity, optical coherence tomography (OCT) revealed bilateral diffuse and severe thinning of the retinal nerve fiber layer (RNFL) in 4 out of 6 patients. Electrocardiograms of all LHON patients were unremarkable.

The mtDNA haplogroups of all LHON patients were varied and did not show any correlation with the presence of the m.13513G > A mutation (Table S2).

### 2.2. Molecular Data

**Figure 2:**
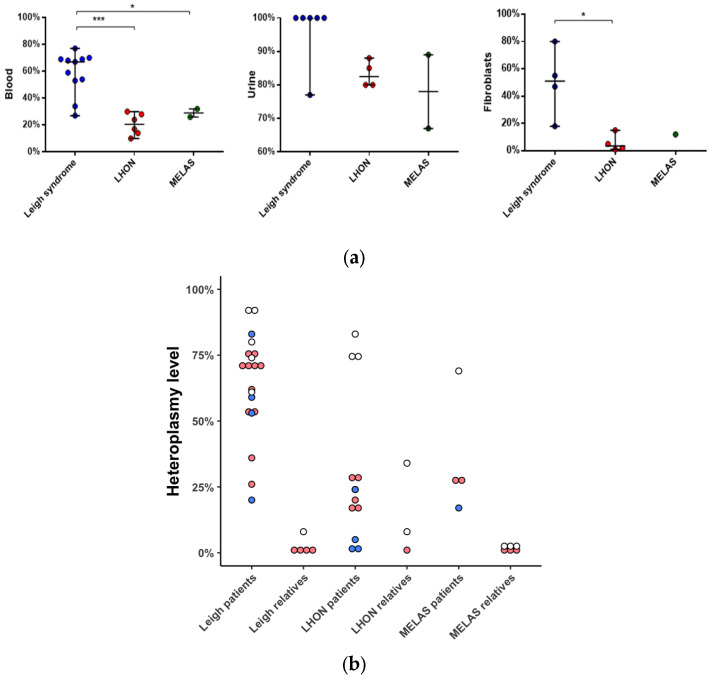
Heteroplasmy level of m.13513G>A in different tissues of patients and their healthy relatives. (**a**) Heteroplasmy level in blood (*n* = 19, NGS); urine (*n* = 12, Sanger); skin fibroblasts (*n* = 9, NGS) of patients. (**b**) Heteroplasmy level in blood (*n* = 27), red; urine (*n* = 15), white; skin fibroblasts (*n* = 9), blue; NGS; of patients and their relatives. *—*p* < 0.05, ***—*p* < 0.0005, Mann–Whitney U-test. Data represents the median with range. NGS—Next Generation Sequencing.

The mutational load varied across tissues and ranged from 10 to 77% (*n* = 19, median value 67%) in blood, from 67 to 100% (*n* = 12, median value 88.5%) in urine, and from 1% to 80% (*n* = 9, median value 15%) in skin fibroblasts (Figure 2). The median mutational load in patients with Leigh syndrome was higher than in those with LHON, both in blood (67 vs. 21%) and in skin fibroblasts (51 vs. 4%).

**Figure 3:**
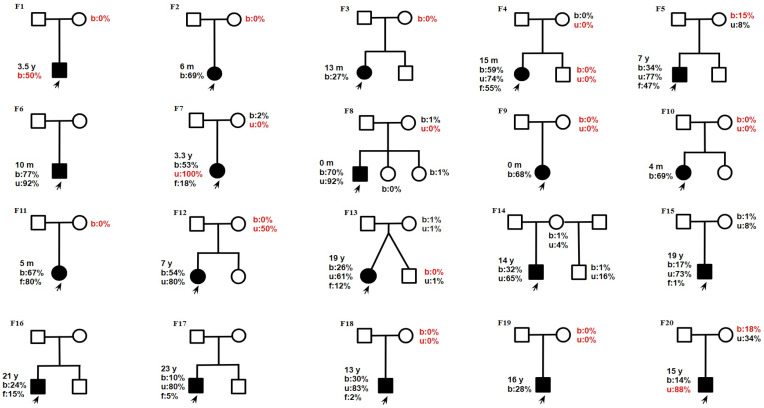
Pedigrees from 20 families. Age of onset: y—year; m—month. Heteroplasmy level b—blood; u—urine; f—skin fibroblasts) revealed by NGS (except data in red which were performed by Sanger sequencing). The proband is marked by a black arrow. Families with F1–F12: Leigh syndrome, F13–F14: MELAS, F15–F20: LHON.

Samples from 17 mothers and 5 siblings were available for validation of the m.13513G>A mutation (Figure 2). In 10 out of 17 cases, m.13513G>A was not detected in blood, but in 1 of these 10 cases, the mutation was validated in a urine sediment sample (F12, mother). In 8 out of 20 cases, m.13513G>A was inherited (Figure 3).

### 2.3. Mitochondrial Bioenergetics

**Figure 4:**
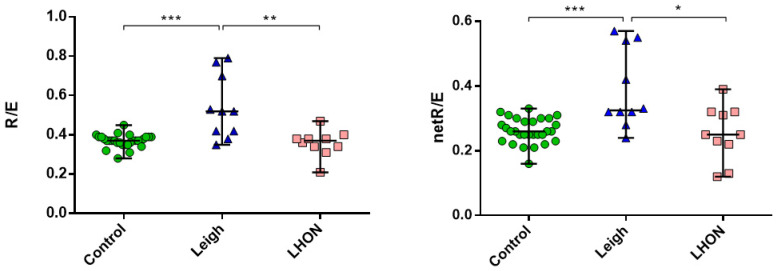
Oxygraphy (Flux Control Ratios, FCRs: R/E and netR/E) on the Oxygraph-2k on intact skin fibroblasts cell lines from LHON (*n* = 4), Leigh (*n* = 4) patients, and control (*n* = 10); ***—*p* < 0.0005, **—*p* < 0.005, *—*p* < 0.05 (vs. control), Mann–Whitney U-test. The data represented as median with range. Experiments were performed in duplicate or triplicate.

We performed high-resolution respirometry on intact fibroblast cell lines from patients (*n* = 8) and controls (*n* = 10) (Figure 4). Significant differences were observed between the Leigh syndrome group versus controls and the LHON group (*p* < 0.0005). The median R/E ratio was 1.4 times higher, and the median netR/E ratio was more than 1.3 times higher in the Leigh group compared to the control group. These results indicate a substantial reduction in maximal respiratory capacity in the Leigh group. No significant differences in Flux Control Ratios (FCRs) were found between the LHON and control groups.

**Figure 5:**
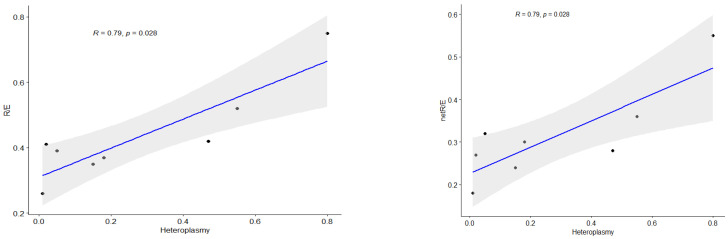
Spearman correlation for non-parametric analysis between mutation load of m.13513G>A and FCR (R/E, netR/E). *R*—Spearman’s correlation coefficient, *p*—*p*-value, blue line—correlation line, grey shading—the 95% confidence interval for the line.

In our cohort, patients with heteroplasmy levels over 55% showed significant differences compared to control data. Figure 5 presents a correlation analysis between the heteroplasmy level in patients’ skin fibroblasts and FCR, showing a strong association between the heteroplasmy level of the m.13513G>A variant and both the R/E and netR/E ratios.

**Figure 6:**
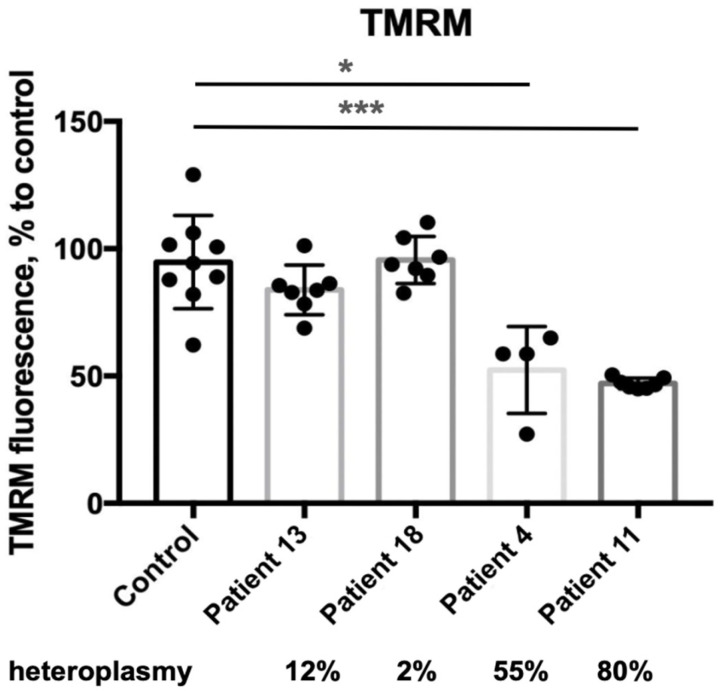
Mitochondrial membrane potential on TMRM (skin fibroblasts). *—*p* < 0.05, ***—*p* < 0.0005 (vs control), Mann–Whitney U-test. Each dot represents an independent experiment, data are presented with the standard deviation.

The mitochondrial transmembrane potential (ΔΨm) of skin fibroblasts with 2% and 12% heteroplasmy was comparable to that of the control group (Figure 6). In contrast, skin fibroblasts with 55% and 80% heteroplasmy exhibited a significant decrease in ΔΨm compared to controls.

## 3. Discussion

The present study included 20 patients with PMD who had a confirmed pathogenic variant, m.13513G>A, in the *MT-ND5* gene, with varying mutation loads. This work has several limitations: the MELAS cohort included two patients, and specific findings associated with this subgroup must be considered. Different nuclear backgrounds may contribute to clinical variability among patients; however, due to insufficient biomaterial, we were unable to perform whole-exome sequencing or whole-genome sequencing in this retrospective cohort.

Globally, mtDNA variants account for 25–31% of all cases of Leigh syndrome [18,19]. In our Leigh cohort, the m.13513G>A variant represents approximately 18% of all mtDNA-related Leigh patients, making it the most prevalent variant among all m.13513G>A cases [19]. Mutation loads for patients with Leigh syndrome ranged from 27 to 77%, which is consistent with global data (40 to 86% for Leigh syndrome and 23 to 59% for Leigh-like syndrome) [12,20,21,22,23,24]. Higher mutation loads were associated with an earlier age of onset; specifically, 4 out of 9 patients with a heteroplasmy level of 66% or higher had an age of onset of less than 6 months. However, our results did not indicate any association between the age of mortality and heteroplasmic load of the m.13513G>A variant. In general, a high level of heteroplasmy is linked to early disease onset in mtDNA-associated Leigh syndrome [12]. While WPW syndrome is a common clinical feature associated with the m.13513G>A variant, it was observed in only 3 patients within our cohort of Leigh syndrome patients [25].

Several studies have reported an association between the m.13513G>A variant and isolated LHON, with heteroplasmy levels ranging from 22.4 to 29% [26,27]. Additionally, there are reports of an LHON and MELAS overlap syndrome and a case involving optic atrophy and nephropathy (7% heteroplasmy) [28,29,30]. Our study presents the most representative cohort of LHON patients (*n* = 6) harboring the m.13513G>A variant, with heteroplasmy levels (detected by Next Generation Sequencing (NGS)) ranging from 10 to 30%. Interestingly, a heteroplasmy level of 10.3% has been recently associated with mitochondrial nephropathy [31]. We did not find evidence of an association between mtDNA haplogroups and increased risk for LHON in patients with the m.13513G>A variant, unlike previous findings related to other mtDNA variants [32].

Furthermore, LHON patients with the m.13513G>A variant tend to have a better long-term prognosis, characterized by spontaneous visual recovery and a milder clinical phenotype, which is also typical for patients carrying the m.14484T>C, m.13094T>C, and m.11253T>C variants [33,34]. In our cohort, LHON patients predominantly presented with low heteroplasmy levels, emphasizing the need for additional testing of DNA from urine sediment to detect the presence of the m.13513G>A variant, especially in laboratories that use Sanger sequencing for routine analysis. The NGS approach is preferable for LHON screening due to its higher sensitivity in detecting low mutation loads [27]. We detected a heteroplasmy level at 1% in 8 patient’s mothers by deep amplicon sequencing, which emphasizes the fact that the variant m.13513G>A was inherited; overwise, it would be missed using just Sanger sequencing, as was shown recently [35].

In our study, several (families F13, F14, F16, F18, F19) patients with MELAS and LHON have the same level of heteroplasmy (less than 40%) in the blood. The mutation load of m.13513G>A for MELAS patients is varied from 11% to 36% and raised with the overlap syndromes: 15–90% for MELAS/Leigh and 50% for MELAS/LHON [11,26,36,37]. To explain the variation between the same heteroplasmy level and phenotypic expression, several publications suggest that it could be defined by different biochemical thresholds in certain tissues and by the heteroplasmy shift during mitotic and meiotic cell division or mtDNA copy number in the tissue [38,39,40]. Further multi-omics investigations are needed to fully understand the mechanism of the phenotypic heterogeneity of m.13513G>A at the same heteroplasmy level [41].

Results of high-resolution respirometry confirmed a significant reduction in maximal respiratory capacity in the cells harboring m.13513G>A from a 55% heteroplasmy level and above. Folmes et al. showed low maximal respiration and reserved capacity in the fibroblast cell lines from MELAS patients (50% heteroplasmy) and three iPCs clones [42]. The correlation analysis results highlight the dependence of respiratory rates on heteroplasmy levels in intact cells. Our data support the previous work of Kidere that there is a threshold of m.13513G>A heteroplasmy between 50 and 62% to induce bioenergetic defects, which result in low oxygen consumption rates [43]. We detected low mitochondrial membrane potential in the fibroblast cell line, harboring m.13513G>A with a 55% heteroplasmy. A significant decrease in the mitochondrial membrane potential was observed in skin fibroblasts with a mutation load of 80% from the Patient 11 with Leigh syndrome, which presented severe clinical features like spastic tetraparesis, status epilepticus, motor regression, dystonia, lesions in medulla oblongata, mesencephalon, and cortex atrophy in the MRI images, WPW syndrome, and anemia. Energy deficiency and redox imbalance caused by complex I dysfunction due to the m.13513G>A pathogenic variant are manifested by a significant reduction in mitochondrial membrane potential. Previous works also reported low-level mitochondrial membrane potential in cells of patients with pathogenic variants in the complex I mtDNA-associated genes [44,45].

Most LHON substitutions (m.3460G>A, m.11778G>A and m.14484T>C) are present in homoplasmy, which implies that those substitutions are mildly deleterious themselves and require additional trigger factors [46]. m.13513G>A has never been reported in the homoplasmic state, and even a low heteroplasmic load could cause different phenotypes, like LHON and mitochondrial nephropathy [29,31]. Asp393 of ND5 is involved in the architecture of the output pathway and placed next to the key proton release cite Lys392; the substitution p.Asp393Asn mostly affects the catalytic function of the ND5 subunit (antiporter-like subunit, essential for proton pumping) in complex I and results in insufficient ATP production, which indicates that m.13513G>A plays an essential role in the OXPHOS system [47,48].

## 4. Materials and Methods

### 4.1. Editorial Policies and Ethical Considerations

This study has been carried out in accordance with The Code of Ethics of the World Medical Association Declaration of Helsinki for experiments involving humans. The work was approved by the local ethics committee of the Federal State Budgetary Institution “Research Centre for Medical Genetics”. The approval number is 4/1 from the 19th of April 2021. Informed consent was obtained from all the studied family members.

### 4.2. Patients and Samples

Twenty unrelated patients (9 females, 11 males) with the m.13513G>A pathogenic substitution, along with their mothers (*n* = 17), were enrolled in this study. Detailed medical records were available for 17 out of 20 patients. Pedigrees are shown in Figure 3.

This study includes DNA samples from the patient’s blood (*n* = 20), urine sediment (*n* = 12), skin fibroblasts (*n* = 9), and also from the patient’s relatives: blood (*n* = 22) and urine sediment (*n* = 16). Samples, including DNA and skin fibroblasts, were deposited at the Moscow Branch of the Biobank “All-Russian Collection of Biological Samples of Hereditary Diseases” (Research Centre for Medical Genetics, Moscow, Russia).

### 4.3. Heteroplasmy Detection

Three different methods were used to assess the heteroplasmy level of the m.13513G>A variant in the studied samples. Due to the absence of biomaterials, for several patients, just Sanger sequencing was provided instead of NGS.

### 4.4. Sanger Sequencing

The heteroplasmy levels in all samples were detected using Sanger sequencing of DNA extracted from the patient’s blood, urine sediment, and skin fibroblasts. The mutation load was measured by determining the peak areas in abi files.

### 4.5. Massive Parallel Sequencing of the Whole mtDNA

DNA samples were extracted from blood cells, urinary sediment epithelium, and skin fibroblasts using a QIAGEN DNA Mini Kit (QIAGEN, Germantown, MD, USA). The entire mtDNA molecule was sequenced using Next Generation Sequencing with two overlapping Long-Range PCR fragments of 8 kb and 9 kb, amplified using High-Fidelity Polymerase (Dialat Ltd., Moscow, Russia). The amplicons were then purified from 1.0% agarose gels using the Monarch DNA Gel Extraction Kit (New England Biolabs, Ipswich, MA, USA) according to the manufacturer’s protocol, or with AMPure XP beads (Beckman Coulter, Brea, CA, USA).

For library preparation, a mix of one or two fragments (each containing 50–100 ng) was used. Libraries were constructed using the Prep&Seq™ G-Fragmentation kit (Parseq Lab, Saint Petersburg, Russia) and sequenced on either the Ion S5 (Life Technologies, Thermo Fisher Scientific, Waltham, MA, USA) or NextSeq 500/MiSeq (Illumina, San Diego, CA, USA) platforms, achieving a coverage depth ranging from ×1376 to ×19,562. The sequencing results were analyzed by aligning them to the Revised Cambridge Reference Sequence (rCRS) NC_012920.1 using IGV software version 2.16.0 (Broad Institute, Cambridge, MA, USA).

GATK MarkDuplicates (picard) tool was used to exclude PCR duplicates in the analysis. The heteroplasmy level was calculated as a ratio (number of reads with pathogenic variant)/(total number of reads in the position 13,513) × 100%.

### 4.6. Amplicon Deep Sequencing

To fine-tune the heteroplasmic load, we performed a two-step PCR approach for the further deep sequencing on the Illumina Platform. In the first step, we used a pair of primers containing a locus-specific sequence around the m.13513 position with a 5′ tail compatible with Prep&Seq™ set («Parseq Lab», Russia) for NGS. The resulting PCR-amplicons were then used as templates within the second-step PCR for further amplification and inclusion of Illumina barcodes and adaptors. The libraries were sequenced on GeneMind («GeneMind Biosciences Co.», Ltd., Shenzhen, China) with the deep coverage from ×187,247 to ×431,272. The heteroplasmy level was calculated as a ratio (number of reads with pathogenic variant)/(total number of reads in the position 13,513) × 100%.

### 4.7. Cell Lines

Primary skin fibroblast cultures were derived from inner forearm skin biopsies of patients (*n* = 9) and healthy volunteers (*n* = 10) using standard fibroblast cell culture techniques. The cells were cultivated at 37 °C until they reached 85% confluence in a specialized proliferative medium called “Amniokar” (PanEco-Ltd., Moscow, Russia).

For the study, the cells were subcultured in DMEM supplemented with 10% FBS and 200 µM uridine. All cell culture and sample preparation services were provided by the Common Use Center “Biobank” at the Research Centre for Medical Genetics in Moscow, Russia.

### 4.8. High-Resolution Respirometry

The skin fibroblasts from patients (*n* = 8) and controls (*n* = 10) were harvested using 0.25% trypsin and centrifuged at 1500× *g* rpm for 5 min. The pellet (1.5−3 × 10^6^ cells/mL) was resuspended in a pre-warmed (at 37 °C) respiration medium MIR05 (100 mM sucrose, 60 mM potassium lactobionate, 0.5 mM EGTA, 3 mM MgCl_2_x·6H_2_O, 20 mM taurine, 10 mM KH_2_PO_4_, 20 mM HEPES, and 1 mg/mL BSA, pH = 7.1).

The analysis of oxygen consumption was performed with the Oxygraph-2k (Oroboros Corp., Innsbruck, Austria) using DatLab 7.0 software. Experiments with intact cells were performed according to the protocol of Pesta and Gnaiger as described previously [14,49]. Flux Control Ratios (FCRs) were used to estimate bioenergetic defects: R/E, L/E, netR/E ((R-L)/E). R—ROUTINE—oxygen flux of living cells in MIR05 without exogenous substrates, L—LEAK—oxygen flux after 2.5 µM oligomycin addition, E—ETS—maximum oxygen flux by stepwise injection of 0.05 μM carbonyl- cyanide p-(trifluoromethoxy) phenylhydrazone (FCCP).

### 4.9. Mitochondrial Membrane Potential

The membrane potential of the mitochondria was assessed as described previously [14].

### 4.10. Statistical Analysis

Statistical analyses and data visualization were performed using GraphPad Prism 6 software and R script in the RStudio program (version 4.3.1). Statistical significance was determined by the non-parametric Mann–Whitney U-test. For all experiments, each control or patient fibroblast cell line was analyzed at least in duplicate (two biological replicates). Correlation analysis was examined using a non-parametric Spearman rank correlation test. All data were considered statistically significant at *p* < 0.05.

## 5. Conclusions

Our results expand the clinical and bioenergetic findings of patients with m.13513G>A-variant-related phenotypes (Leigh syndrome, MELAS, LHON) with varying heteroplasmy levels (from 10 to 77% in blood). We did not observe dependance on the heteroplasmy level and phenotype of PMD, but we emphasized that a high heteroplasmic level (more than 67%) is associated with early manifestation. We suggest a threshold of 55% of heteroplasmy in fibroblast cell lines to lead to a bioenergetic dysfunction like high oxygen consumption rates and low mitochondrial membrane potential. Our data suggest that even a slight shift in heteroplasmy can improve cellular function and, consequently, the patients’ phenotype, providing a solid foundation for the development of future gene therapies for mtDNA diseases.

## Data Availability

Data is contained within the article and Supplementary Materials.

## Supplementary Materials

The following supporting information can be downloaded at https://www.mdpi.com/article/10.3390/ijms26104565/s1.

## Abbreviations

The following abbreviations are used in this manuscript:

PMD	Primary mitochondrial disorder
mtDNA	Mitochondrial Deoxyribonucleic acid
MELAS	Mitochondrial Encephalomyopathy with Lactate Acidosis and Stroke-like episodes
LHON	Leber’s Hereditary Optic Neuropathy
OXPHOS	Oxidative phosphorylation
DNA	Deoxyribonucleic acid
