# Supplementary material for: Exploring the Phenotypic Heterogeneity and Bioenergetic Profile of the m.13513G>A mtDNA Substitution: A Heteroplasmy Perspective
Source: Int J Mol Sci. 2025 May 10;26(10):4565. doi: 10.3390/ijms26104565 (PMC12111569; doi:10.3390/ijms26104565)
Supplement: Supplementary file 1 [file ijms-26-04565-s001.zip › Supplementary table S2.pdf]

Supplementary Table S2. *Clinical data of 6 male unrelated LHON patients with m.13513G>A variant. N/A - not available.*

Patient	***Patient 15 OD/OS***	***Patient 16 OD/OS***	***Patient 17 OD/OS***	***Patient 18 OD/OS***	***Patient 19 OD/OS***	***Patient 20 OD/OS***
Age at onset (years)	*19,4/19,4*	*21,6/21,6*	*23,3/23,3*	*13/13,1*	*16,3/16,2*	*15/15*
Time to involvement of second eye (weeks)	*0*	*0*	*2*	*3*	*2*	*0*
Visual acuity at nadir (decimal)	*0,01/0,005*	*0,08/0,01*	*0,03/0,02*	*0,02/0,03*	*0,005/0,01*	*0,02/0,03*
Visual acuity at last visit (decimal)	*1,0/1,0*	*0,8/1,0*	*0,3/0,7*	*0,8/1,0*	*N/A*	*0,1/0,1*
Color blindness test (27 tables)	*7/5*	*11,5/11,5*	*7/5,5*	*9/12*	*N/A*	*5/8,5*
Average RNFL OD/OS; Average GCC OD/OS (µm)	*66,20/54,56; 46,98/48,25*	*59,39/55,49; 50,38/52,07*	*57,17/58,60; 58,60/61,48*	*58,43/55,96; 52,7/53,6*	*N/A*	*N/A*
Haplogroup	*H5e1a*	*W1*	*T2b2b1*	*HV0+195*	*H1e2*	*D4b1a2a*
